# Immigrant Background and Rape Conviction: A 21-Year Follow-Up Study in Sweden

**DOI:** 10.1177/08862605241311611

**Published:** 2025-01-06

**Authors:** Ardavan M. Khoshnood, Jan Sundquist, Kristina Sundquist

**Affiliations:** 1 Emergency Medicine, Department of Clinical Sciences Malmö, Lund University, Malmö, Sweden; 2 Center for Primary Health Care Research, Department of Clinical Sciences Malmö, Lund University, Malmö, Sweden

**Keywords:** rape, Sweden, integration, case-control study, acculturation

## Abstract

While extensive research exists on the severe consequences among rape victims, little is known about specific predictors in relation to rape convictions among immigrants to Europe. This study from Sweden (having one of Europe’s highest per capita rates of rape) investigates individuals convicted of rape, aggravated rape, attempted rape, or attempted aggravated rape, collectively termed as rape+, against women 18 years or older, from 2000 to 2020. In this case-control study, we analyzed data from Swedish population-based registers. The analysis includes 4,032 individuals convicted of rape+ and 20,160 matched controls. We used logistic regression models to examine the relationship between immigrant background and rape+ convictions, while adjusting for several potential confounders. We found that 36.9% of the convicted individuals and 69.5% of the controls were Swedish-born with two Swedish-born parents. The odds of being convicted of rape were higher for individuals with an immigrant background across all models. After adjusting for potential confounders (socioeconomic status, substance use disorders, psychiatric disorders, and criminal behavior), these odds decreased but remained significant, especially for those born outside Sweden and arriving at age 15 or older. Our findings reveal a strong link between immigrant background and rape convictions that remains after statistical adjustment. The mechanisms behind the overrepresentation of individuals with an immigrant background among those convicted of rape+ need further exploration.

## Background

Rape may lead to severe consequences among the victims, such as somatic complications and disabilities as well as long-term psychiatric disorders (PD) (Campbell et al., 2003; Koss et al., 1994; Nadelson, 1989). The significant impact of rape on the victims underscores the necessity for further research to acquire new insights that can enhance preventive efforts in healthcare, social services, and criminal institutions. Understanding the specific predictors contributing to a potentially heightened risk of committing rape is, therefore, of utmost importance in the assessment of potential sexual offenders (Mann et al., 2010).

Sweden is currently struggling with increasing rates of violent crimes. While the country has witnessed a modest increase in the rate of deadly violence and a significant increase in gang-related gun violence, it also registers the highest per capita rate of rapes in Europe (Holmberg & Lewenhagen, 2020; Khoshnood, 2018). Although parts of the high rate of rapes can be related to changes in the legislation and the method of statistical recording (Holmberg & Lewenhagen, 2020), it is still vital to examine available predictors for rape in a broad perspective based on nationwide data with a long study period rather than on small, local samples.

In the current study, we aimed to investigate predictors in all individuals in Sweden convicted of rape, aggravated rape, attempted rape, or attempted aggravated rape, collectively referred to as rape+, against women who were 18 years or older, spanning the years 2000 to 2020. Examining the characteristics of potential offenders is crucial for enhancing risk analysis, risk assessments, and prevention efforts across healthcare, social services, and criminal institutions.

### Swedish Legislation and Statistics

Chapter 6 of the Swedish Criminal Code (1962:700), addressing sexual offences against both adults and minors, has undergone numerous modifications since its inception in 1965. It has been amended a total of six times, with the most recent alteration occurring in 2018. These changes have broadened the definition of rape, lowering the necessity for physical force as an element of the crime. Today, the Swedish legal system has made a landmark shift toward consent-based legislation, culminating in the introduction of “negligent rape” (Wegerstad, 2021).

In 2022, the Swedish police received 4,810 reported instances of rape against adult women, aged 18 or older (Brottsförebyggande rådet, 2023a). However, only 325 individuals were convicted of rape offences during the same time period (Brottsförebyggande rådet, 2023b). This substantial difference presents a major problem for the police and the prosecution, as it brings up the challenge of proving that a rape has actually occurred (Holmberg & Lewenhagen, 2019).

Irrespective of the statistical and legal interpretations behind the high reported rates, it is undeniable that sexual offences, particularly rape, pose a serious problem in Sweden. As indicated by the National Security Study of 2022 conducted annually by the Swedish National Council for Crime Prevention (In Swedish: Brottsförebyggande rådet, shortened BRÅ), 10% of the Swedish populace frequently or somewhat frequently express fears of becoming victims of rape or other sexual abuse. As might be expected, a larger proportion of women (18%) compared to men (2%) express concerns about falling prey to sexual crimes, encompassing rape (Holst et al., 2022).

### Swedish Rape Offenders

While there exists a considerable body of research examining rape in Sweden from a legal perspective (e.g., Bladini & Andersson, 2020; Von Hofer, 2000; Wegerstad, 2021), as well as studies centered on Swedish rape victims (e.g., Ceccato et al., 2020; Rudolfsson, 2024; Tiihonen Möller et al., 2014), the scholarly literature on Swedish rape offenders remains scarce. Most Swedish studies on rape offenders have focused on their relationship with the victim, as well as the sex and age of the offenders (Grevholm et al., 2005; Hradilova Selin, 2008).

There are, however, numerous international studies on risk factors for violent crimes including rape (e.g., Gottfredson & Hirschi, 1990; Holmes & Holmes, 2009; Kocsis, 2010; Wikström & Sampson, 2009). Knight and Prentky (1987) divided rapists in four different typologies and asserted that some of the most common risk factors were alcohol abuse, PD, and criminal behavior (CB). Other important risk factors are low socioeconomic status, neighborhood disadvantage, belonging to a certain ethnicity, male sex, and drug abuse (Hirschi, 2009; Raine, 2013). In a comprehensive review on risk and protective factors for sexual offending, Seto et al. (2023) identified several risk factors including hostile masculinity and negative peer influence, alongside protective factors such as supportive social and professional networks, and engagement in employment or constructive leisure activities. The authors also highlighted the importance of cultural and ethnic differences in these factors, emphasizing the need for further studies to ensure the generalizability of research findings across diverse populations.

One previous study profiled 21 male offenders convicted of rape or aggravated rape against adult females in Malmö, Sweden, from 2013 to 2018. The authors of that study stated that these 21 male offenders were on average 33 years old, usually single, and often of foreign origin (Stiernströmer et al., 2020).

### The Current Study

To the best of our knowledge, no previous study has examined potential confounders and the abovementioned predictors’ (socioeconomic status, substance use disorder, PD, as well as CB) importance in the association between immigrant status and rape+, which will be done in the current study. We also hypothesized that lack of acculturation and integration are important contributing factors in these associations and included subgroups and covariates that may reflect a person’s acculturation and integration in the society.

Acculturation and integration are both complex features that are partly related to each other, and the measures in the current study should only be considered as proxies of these features. Acculturation refers to the adaptation that takes place in immigrants when they are exposed to a new culture, and immigrants’ different acculturation strategies can include both negative and positive aspects. These have been defined in Berry’s model of acculturation as integration, assimilation, separation, and marginalization, and the concepts of acculturation have been widely used after they were first minted by Berry (1980). A systematic review from 2021 included 21 primary studies, which assessed 61,885 migrants, and found that marginalization and separation were associated with worse depression and anxiety-related symptoms compared to integration (Choy et al., 2021). The systematic review also identified that acculturation stress can be aggravated by poor socioeconomic conditions, which are also related to integration (Fleischmann & Dronkers, 2010). The OECD (2019) emphasized in a report on integration and migration the importance to “organize resources to reduce the influence of socio-economic status on the outcomes of immigrants” in the road to integration.

The present study included several socioeconomic measures as well as two established measures of acculturation in first-generation immigrants, that is, age at arrival and time lived in the new country. Previous research has also found differences in acculturation strategies between first-generation immigrants and their children (i.e., second-generation immigrants) who were also included in the present study (Cobb et al., 2021).

Our objectives were as follows:

1. The first aim was to examine the odds of being convicted of rape among men with an immigrant background; these men were further subdivided into the categories born outside Sweden (first-generation immigrants) and born in Sweden (second-generation immigrants). The analyses also considered age at arrival and years lived in Sweden (first-generation) as well as whether one or both parents were born outside Sweden (second-generation).
2. The second aim was to control these potential associations for socioeconomic factors, substance use disorders, and PD as well as CB, which are all considered as predictors for rape conviction.

This study represents a novel contribution because we consider previously identified predictors (including several socioeconomic measures) and also include potential measures of acculturation such as being born in Sweden or abroad and, in the latter subgroup, age at arrival to Sweden and years lived in Sweden. Although our measures of acculturation are not direct measures of integration, they are quite established measures of acculturation, which in turn includes the concept integration as one possible result.

Our study is important because very little research exists on this sensitive topic and, under these circumstances, such knowledge and facts must be presented by independent researchers from established research institutions.

## Methods

We analyzed information on individuals from Swedish population-based registers with national coverage. These registers were granted to us from the National Board of Health and Welfare, Statistics Sweden, and the Swedish National Council for Crime Prevention. The individual-level data were linked using each individual’s unique identification number replaced by a serial number to preserve confidentiality. We secured ethical approval for this study from the Swedish Ethical Review Authority (Dnr 2021-04268).

The database was created by selecting all individuals convicted of rape+ against a woman between 2000 and 2020 and who were between 15 and 60 years old. The lower cut point of 15 years of age was selected since a person is not legally responsible for a committed crime in Sweden before the age of 15. The upper age limit was restricted because we only had access to data on individuals born 1958 and younger.

Our focus on female victims is partly because women are more common rape victims than men and partly because rape offenders have different profiles depending on the characteristics of the victims (Simons, 2017). In total, we investigated 4,032 unique individuals. Rape+ were defined as stated in the Swedish Criminal Code (1962:700). Their penal code chapters and sections, as well as the crime codes as defined by the Swedish Brå, were as follows: Rape and aggravated rape, Chapter 6, § 1; BRÅ codes: 9639, 9635, 9640, 9636, 9641, 9637, 9642, 9638; Attempted rape and attempted aggravated rape, Chapter 6, § 15; BRÅ codes: 9631, 9627, 9632, 9628, 9633, 9629, 9634, 9630 (Brottsförebyggande rådet, 2018).

We used a national matched case-control design where all individuals convicted of rape+ were matched for sex and year of birth to five individuals randomly sampled from the general population and who were living in Sweden on the individual’s conviction date (the index date). This matched design enables assignment of an index date to individuals without convictions (the control group) in an unbiased manner. In total, the 4,032 individuals were matched to 20,160 individuals who were not convicted of rape. In the database, we included the following variables (see Supplemental Appendices 1 and 2 for more details): immigrant status (i.e., born in Sweden with one parent born outside Sweden, born in Sweden with two parents born outside Sweden and born outside Sweden). We also included age at arrival and years lived in Sweden (for those born outside Sweden), social welfare recipient, neighborhood deprivation status, and income. The socioeconomic variables were measured the year prior to registration for rape+. Furthermore, registrations for alcohol use disorder (AUD), drug use disorder (DUD), PD, and CB were included in the database. These registrations could occur any time prior to time of registration for rape+. CB was defined as any registration in the Swedish criminal registers. The included variables could be regarded as “predictors” for rape although we only had access to data on rape convictions rather than the actual rates of committed rapes.

We performed four consecutive logistic regression models with a separate stratum for each case and their controls (Tables 2 and 3). In Model A, we only included immigrant background categorized into five groups: born in Sweden to Swedish born parents (reference in the analysis), born in Sweden with one parent born outside Sweden, born in Sweden with two parents born outside Sweden, born outside Sweden who arrived at age 15 or older, and born outside Sweden who arrived before age 15. In Model B, we additionally included the variables social welfare recipient, neighborhood deprivation status and income, while model C further included AUD, DUD, and PD. The final model was additionally controlled for CB. In an additional regression analysis, we categorized immigrant status slightly different. The group born outside Sweden was here categorized into three groups (more than 10 years in Sweden, 5–10 years in Sweden, 0–5 years in Sweden), leading to a six-category definition of immigrant background. All statistical calculations were performed using SAS 9.4 (SAS Institute).

## Results

Table 1 shows the cases and the age- and sex-matched controls by the included covariates. Our study included 4,032 cases and 20,160 matched controls of which 99.7% were males. The mean birth year was 1982 (*SD* = 11 years).

**Table 1.** Number and Percentages of Individuals Convicted of Rape (Cases) Between 2000 and 2020, Born Between 1960 and 2004 and Matched to 5 Controls Based on Year of Birth and Sex.

Variables	Cases	Controls
*n*	4,032	20,160
Mean year of birth (*SD*); range	1982 (11); 1960–2004	
Males	4,019 (99.7%)	
Born in Sweden, 2 Swedish-born parents	1,488 (36.9%)	14,016 (69.5%)
Born in Sweden, 1 parent born outside Sweden	247 (6.1%)	1,549 (7.7%)
Born in Sweden, 2 parents born outside Sweden	256 (6.4%)	976 (4.8%)
Born outside Sweden	2,041 (50.6%)	3,619 (18.0%)
(A1) Born outside Sweden arrived <15 years of age	666 (16.5%)	1,377 (6.8%)
(A2) Born outside Sweden arrived ≥15 years of age	1,375 (34.1%)	2,242 (11.1%)
(B1) Born outside Sweden (>10 years in Sweden)	715 (17.7%)	1,546 (7.7%)
(B2) Born outside Sweden (5–10 years in Sweden)	398 (9.9%)	750 (3.7%)
(B3) Born outside Sweden (<5 years in Sweden)	928 (23.0%)	1,323 (6.6%)
Social welfare^a^	1,415 (35.1%)	1,871 (9.3%)
Neighborhood deprivation^a^	1.57 (2.5)	0.53 (2.1)
Income (100,000 Swedish Krona)^a^	1.23 (0.9)	1.66 (1.3)
Alcohol use disorder^b^	601 (14.9%)	653 (3.2%)
Drug use disorder^b^	955 (23.7%)	1,020 (5.1%)
Psychiatric disorder^b^	523 (13.0%)	790 (3.9%)
Criminal behavior^b^	2,096 (52.0%)	2,709 (13.4%)

a Measured the year prior to conviction for rape.

b Measured any time prior to date of conviction for rape.

Considering origin, 36.9% (*n* = 1,488) of the case group and 69.5% (*n* = 14,016) of the control group were born in Sweden to two Swedish-born parents. In total, 12.5% (6.1% + 6.4%) among the cases were born in Sweden and had at least one parent born outside Sweden and 50.6% were born outside Sweden (Table 1). Among the controls, 18.0% were born outside Sweden. Those born outside Sweden were further subdivided by age at arrival (A1, A2) and years lived in Sweden (B1, B2, B3). Regarding age at arrival for those born outside Sweden, a larger proportion of the case group arrived in the country at age 15 years or older (*n* = 1,375; 34.1%) than those who arrived prior to the age of 15 (*n* = 666; 16.5%). In the control group, the corresponding proportions were 11.1% (*n* = 2,242) and 6.8% (*n* = 1,377), respectively.

For the variable years lived in Sweden for those born outside the country (in total 50.6% among the cases), 23.0% (*n* = 928) had lived in Sweden less than 5 years.

A higher proportion in the case group (*n* = 1,415; 35.1%) received social welfare compared to the control group (*n* = 1,871; 9.3%). The case group also had a higher mean neighborhood deprivation score than the control group and lower income levels.

In terms of psychiatric and behavioral disorders, the case group had higher rates of AUD (14.9% vs. 3.2%), DUD (23.7% vs. 5.1%), PD (13.0% vs. 3.9%), and CB (52.0% vs. 13.4%) compared to the control group.

Table 2 shows the results from the conditional logistic regression as odds ratio (ORs) with 95% confidence intervals (CIs) where those born in Sweden with two Swedish-born parents are used as reference in Model A. Those born outside Sweden are further subdivided by age at arrival. The results show higher odds of being convicted of rape among those born outside Sweden and those born in Sweden with one or two parents born outside Sweden across all models when compared to the reference group. The statistically significant ORs for those individuals born in Sweden with one parent born outside Sweden; born in Sweden with two parents born outside Sweden; born outside Sweden and arrived prior to age 15; and born outside Sweden and arrived at age 15 or older were 1.50 (95% CI = [1.31, 1.72]), 2.44 [2.14, 2.79], 4.57 [4.16, 5.03], and 6.25 [5.76, 6.78], respectively, in Model A. In Model B, after adjustment for the socioeconomic factors, the ORs decreased but remained significant. For example, for those born outside Sweden who arrived at age 15 or older, the OR was 3.14 [2.84, 3.48]. After further adjustments for psychiatric and substance use disorders (Model C) and CB (Model D), the ORs decreased further in almost all subgroups and no longer remained statistically significant for those individuals born in Sweden with one parent born outside Sweden (OR 1.12 [0.95, 1.32]). However, among those born outside Sweden and who arrived at age 15 or older, the ORs increased after the adjustments to 4.64 [4.17, 5.16] and 6.22 [5.54, 6.99] in Models C and D, respectively. CB had a particularly high odds of being convicted of rape when all factors were accounted for in Model D (OR = 5.72 [5.21, 6.28]).

**Table 2.** Conditional Logistic Regression on Individuals Convicted of Rape Between 2000 and 2020 and Matched to 5 Controls Based on Year of Birth and Sex.

Variables	Model A	Model B	Model C	Model D
Born in Sweden, 2 Swedish-born parents	Reference	Reference	Reference	Reference
Born in Sweden, 1 parent born outside Sweden	1.50 [1.31, 1.72]	1.31 [1.14, 1.51]	1.22 [1.05, 1.42]	1.12 [0.95, 1.32]
Born in Sweden, 2 parents born outside Sweden	2.44 [2.14, 2.79]	1.74 [1.49, 2.03]	1.61 [1.36, 1.91]	1.49 [1.24, 1.78]
Born outside Sweden arrived <15 years of age	4.57 [4.16, 5.03]	2.74 [2.45, 3.07]	2.80 [2.49, 3.15]	2.37 [2.09, 2.67]
Born outside Sweden arrived ≥15 years of age	6.25 [5.76, 6.78]	3.14 [2.84, 3.48]	4.64 [4.17, 5.16]	6.22 [5.54, 6.99]
Social welfare		3.53 [3.23, 3.86]	2.59 [2.36, 2.85]	2.03 [1.84, 2.24]
Neighborhood deprivation		1.05 [1.03, 1.07]	1.04 [1.02, 1.06]	1.03 [1.01, 1.05]
Income		0.81 [0.76, 0.86]	0.90 [0.85, 0.96]	0.96 [0.91, 1.01]
Alcohol use disorder			2.79 [2.39, 3.26]	2.04 [1.73, 2.39]
Drug use disorder			3.47 [3.08, 3.91]	1.97 [1.72, 2.24]
Psychiatric disorder			2.23 [1.93, 2.58]	1.83 [1.58, 2.13]
Criminal behavior				5.72 [5.21, 6.28]

*Note*. Odds ratios and 95% CIs. Those born outside Sweden are further subdivided by age at arrival.

Table 3 shows the results from the conditional logistic regression as ORs with 95% CIs where those born in Sweden with two Swedish-born parents are used as reference in Model A. Those born outside Sweden are further subdivided by years in Sweden. The results from this regression analysis, which considered years in Sweden, showed that the ORs for being convicted of rape were consistently higher among those born outside Sweden and those born in Sweden with one or two parents born outside Sweden across all models when compared to the reference group. The highest odds in Model A were noted for those born outside Sweden and residing in the country for less than 5 years (OR = 7.11 [6.50, 7.78]). The inclusion of socioeconomic factors, psychiatric and behavioral disorders (Models B, C, and D) resulted in a decrease in these ORs that, however, remained consistently elevated in almost all subgroups but no longer remained statistically significant in model D for those individuals born in Sweden with one parent born outside Sweden (OR = 1.13 [0.96, 1.34]). However, among those born outside Sweden and who had resided in the country for less than 5 years, the ORs increased after the adjustments to 4.78 [4.23, 5.40] and 6.90 [6.05, 7.87] in Models C and D, respectively.

**Table 3.** Conditional Logistic Regression on Individuals Convicted of Rape Between 2000 and 2020 and Matched to 5 Controls Based on Year of Birth and Sex.

Variables	Model A	Model B	Model C	Model D
Born in Sweden, 2 Swedish-born parents	Reference	Reference	Reference	Reference
Born in Sweden, 1 parent born outside Sweden	1.51 [1.32, 1.73]	1.31 [1.14, 1.51]	1.23 [1.05, 1.43]	1.13 [0.96, 1.34]
Born in Sweden, 2 parents born outside Sweden	2.48 [2.17, 2.84]	1.75 [1.50, 2.04]	1.65 [1.39, 1.95]	1.55 [1.30, 1.86]
Born outside Sweden (>10 years in Sweden)	4.45 [4.05, 4.90]	2.88 [2.58, 3.21]	3.10 [2.77, 3.47]	2.73 [2.42, 3.08]
Born outside Sweden (5–10 years in Sweden)	5.29 [4.71, 5.96]	2.93 [2.55, 3.38]	3.84 [3.31, 4.44]	4.44 [3.78, 5.22]
Born outside Sweden (<5 years in Sweden)	7.11 [6.50, 7.78]	3.14 [2.79, 3.53]	4.78 [4.23, 5.40]	6.90 [6.05, 7.87]
Social welfare		3.51 [3.21, 3.84]	2.53 [2.30, 2.78]	1.94 [1.76, 2.14]
Neighborhood deprivation		1.05 [1.03, 1.07]	1.04 [1.02, 1.06]	1.04 [1.02, 1.06]
Income		0.81 [0.76, 0.86]	0.90 [0.85, 0.96]	0.96 [0.91, 1.02]
Alcohol use disorder			2.73 [2.34, 3.18]	1.96 [1.73, 2.25]
Drug use disorder			3.44 [3.05, 3.87]	1.97 [1.73, 2.25]
Psychiatric disorder			2.21 [1.91, 2.56]	1.81 [1.56, 2.10]
Criminal behavior				5.64 [5.14, 6.19]

*Note*. Odds ratios and 95% CIs. Those born outside Sweden are further subdivided by years lived in Sweden.

## Discussion

The current study embarks on an exploration of several determinants correlated with rape convictions within Sweden, focusing predominantly on the influence of immigrant background. A pronounced connection between immigrant background and the propensity for rape conviction was unveiled, partly diminishing but persisting even after adjustments for various socioeconomic aspects, substance abuse, PD, and CB. This association was notably pronounced among individuals born outside Sweden and residing within the country for a time span less than 5 years and/or those who arrived at 15 years of age and older, suggesting a possible role of acculturation in deciphering these findings (Andersen & Tranæs, 2011; Långström, 2004; Skardhamar et al., 2014). In addition, we also noted a suggested gradient where the odds increased from the lowest odds among those who were born in Sweden with one foreign-born parent, followed by those who were born in Sweden with two foreign-born parents and with the highest odds among those who were foreign-born in these associations. It is, however, important to keep in mind that our results are representative only for the time period studied in the present analyses. In addition, Sweden has 10 million inhabitants where just above 2 million are born outside of Sweden and most of these immigrants will not commit or be convicted of rape.

Historical data have delineated the overrepresentation of foreign-origin individuals in Sweden as suspects and convicts in criminal activities, which is why our findings of an overrepresentation of immigrants among those convicted of rape are not new. The Swedish National Council for Crime Prevention presented in 2021 a broad report on all types of crimes and found a strong overrepresentation among people with an immigrant background (Brottsförebyggande rådet, 2021). However, the specific causative factors driving this trend remain inadequately explored (Ahlberg, 1996; Brottsförebyggande rådet, 2021; Martens & Holmberg, 2005), which calls for further studies in order to develop efficient preventive policies. The present study was also unable to determine the specific causative factors behind our findings although the observed associations indicate the predictors that will require a future development of tailored preventive policies.

It could be argued that a possible explanation behind our findings involves potential biases present within the law enforcement and judiciary systems. Although this concept remains contested, existing literature has suggested the possibility of such biases (Andersen et al., 2017; Sarnecki, 2006; Skardhamar et al., 2014; Sommers, 2007). However, biases within the law enforcement and the judiciary system cannot explain the substantial overrepresentation of immigrants in the criminal statistics.

Although the concept of integration is not easily defined and represents a complex societal process, a conceivable rationale behind the overrepresentation of crime among immigrants may reside in the shortcomings in integration of immigrants in many European countries. This may potentially culminate in various detrimental consequences, encompassing PD and criminal conduct (Andersen & Tranæs, 2011; Killias, 1997; Manatschal & Stadelmann-Steffen, 2013; Skardhamar et al., 2014).

While immigrant background emerged as a salient variable, the findings of our study also shed light on the complex interconnection with social welfare receipt, neighborhood deprivation, income, and psychiatric and behavioral disorders (Table 1). The augmented proportion of cases receiving social welfare and manifesting elevated rates of AUD, DUD, PD, and CB, juxtaposed with the control group, suggest their close linkage with integration challenges (Ahlberg, 1996). Previous studies have demonstrated that factors such as education, employment, and cultural barriers can predispose immigrants to criminal activities (Killias, 1997; Skardhamar et al., 2014). While employment sometimes (but not always) ensures a successful integration (Kogan, 2016), it is important to keep in mind that socioeconomic factors cannot solely explain the increased risks of criminal activities. A comprehensive review by the Swedish Brå suggests that the current literature establishes only a marginal correlation between socioeconomic status and CB (Ring & Shannon, 2023). A contemporaneous report from the Finnish government corroborates this, noting that although immigrants may display a heightened likelihood of perpetrating sexual offenses, this elevated risk cannot be solely attributed to social disadvantage (Vauhkonen et al., 2021) although socioeconomic characteristics should be considered. A Swedish study of the familial aggregation of sexual crime rates among fathers and brothers of sexual offenders found that genetic factors (40%) and nonshared environmental factors (58%) explained the liability to offend to a higher extent than shared environmental influences (2%) (Långström et al., 2015).

Our stepwise analytic models showed a substantial importance of socioeconomic characteristics where the increased risks among those who arrived at age 15 or older decreased from 6.25 [5.76, 6.78] to 3.14 [2.84, 3.48] after inclusion of the socioeconomic factors. This means that socioeconomic factors should not be neglected in the exploration of potential causative factors behind the increased risks of criminal activities, such as rape, in immigrants. Furthermore, CB was the strongest predictor of rape+ conviction in the final model, indicating that individuals with a history of CB have significantly higher odds of being convicted of rape. This is in line with other studies which emphasize prior criminality as one of the most important risk factors for committing further crimes inclusive rape+. Together with History of Antisocial Behavior (i.e., prior criminality), Antisocial Personality Pattern, Antisocial Cognitions, and Antisocial Associates constitute what Andrews and Bonta called the “Big Four,” which are considered to be major risk factors of CB (Bonta & Andrews, 2010).

Interestingly, our results also showed that for those being born outside of Sweden and being 15 years of age or older when entering the country, the ORs increased in our models after the adjustments. This may be because, for these individuals, the data assessments were limited to a shorter time period.

There are also several other potential underlying factors behind our findings that we, unfortunately, were unable to examine, such as detailed familial characteristics, circumstances during the upbringing, traumatic exposures, peer pressure, exposure to criminal milieus, and cultural factors that further complicate our conclusions. In a study conducted in Kerala, India, where 37 young men were interviewed, it was found that their attitudes toward rape were significantly influenced by their perceptions of sex (Reshma et al., 2022). This viewpoint is deeply anchored in societal norms that sometimes misrepresent or misunderstand women’s roles and rights. The influence of Western culture, especially on women’s clothing choices, was a notable point of discussion. A dominant sentiment among the participants was that the influx of Western practices and cultures might be a catalyst for sexual violence. Provocative body language and attire, often associated with Western culture, were identified as potential triggers. Given that the sample size consisted of only 37 individuals, it is crucial to interpret these previous findings with caution, recognizing the potential limitations in generalizing the results to the views of a broader population.

Numerous studies underpin the importance of sociocultural factors in sexual aggression and sexual crimes (Kalra & Bhugra, 2013). Stermac et al. (1990) emphasize that sexual aggression’s historical pervasiveness is deeply rooted in societal processes and attitudes. For instance, prior studies have shown that societies characterized by patrilocality (i.e., the woman moves in to or close to the husbands’ family) and high levels of feuding are more prone to have high rates of rape, and that certain societies that endorse ideologies of male dominance and female inferiority show a correlation with increased sexual aggression (Quinsey, 1984; Sanday, 1981). As immigrants navigate in their new country, they might be disproportionately influenced by these cultural factors, potentially leading to a heightened inclination toward committing sexual crimes. However, our findings also suggest that the increased risks may decline over time, that is, when a successful acculturation and integration occurs.

The limitations of our study include a lack of valid data on integration and cultural factors, including language spoken at home that is a well-established measure of acculturation. To achieve better knowledge on the potential influence of these factors, other types of methodologies are needed, such as questionnaires and in-depth interviews using qualitative methods. Another important limitation is that residual confounding could have been present. For example, we were unable to fully assess the potential confounding of psychiatric and substance use disorders as well as other types of crime in those individuals who had arrived in Sweden at a higher age. This is because the assessment period was shorter than among those who had lived in Sweden for a longer period. For those individuals in our study population who had been in Sweden for a shorter period, we were unable to adjust our models as robustly as for the other subgroups and that represents a limitation. Furthermore, the coding of certain variables, such as PD and CB, does not account for the variations in types or severity of these disorders. Specifically, PD and CB were coded dichotomously, without distinguishing between the different types of disorders or single versus repeated crimes. This simplification may have overlooked the stronger associations that certain disorders or repeated crimes might have with rape convictions. Finally, due to the use of nationwide register data, our study was unable to measure all aspects of diversity, such as gender identity, sexual orientation, race, ethnicity, language, religion, and culture although many other aspects of diversity were considered, that is, age, sex (only 0.3% of the sample were women), socioeconomic status, nationality, and geography. This means that our findings cannot be generalized to all groups in society. However, these limitations were balanced out by many strengths. Due to the personal identification number, replaced by a pseudonymized serial number, it was possible to link socioeconomic data to clinical diagnoses and criminal convictions as well as information on each individual’s parents. The personal identification number also minimized any loss to follow-up.

## Conclusion

Our findings, based on several different types of immigrant subgroups, show strong associations with the odds of rape conviction in some of these subgroups, even after adjustment for several covariates (socioeconomic status, substance use disorders, PD, and CB). The results also suggest a potential influence of acculturation and integration on the odds of rape. It is, however, important to note that our study only used crude proxies of these complex features and our findings could therefore be regarded as hypothesis-generating, which could encourage many Western societies to further examine whether acculturation and integration policies that are not only limited to socioeconomic factors could be improved. Furthermore, our findings could create a basis for future studies on how preventive policies can be improved in healthcare, social services, and criminal institutions.

## Supplemental Material

sj-doc-2-jiv-10.1177_08862605241311611 – Supplemental material for Immigrant Background and Rape Conviction: A 21-Year Follow-Up Study in Sweden Supplemental material, sj-doc-2-jiv-10.1177_08862605241311611 for Immigrant Background and Rape Conviction: A 21-Year Follow-Up Study in Sweden by Ardavan M. Khoshnood, Jan Sundquist and Kristina Sundquist in Journal of Interpersonal Violence

sj-docx-1-jiv-10.1177_08862605241311611 – Supplemental material for Immigrant Background and Rape Conviction: A 21-Year Follow-Up Study in Sweden Supplemental material, sj-docx-1-jiv-10.1177_08862605241311611 for Immigrant Background and Rape Conviction: A 21-Year Follow-Up Study in Sweden by Ardavan M. Khoshnood, Jan Sundquist and Kristina Sundquist in Journal of Interpersonal Violence
