# Supplementary material for: Immigrant Background and Rape Conviction: A 21-Year Follow-Up Study in Sweden
Source: J Interpers Violence. 2025 Jan 6;41(1-2):372–90. doi: 10.1177/08862605241311611 (PMC12662818; doi:10.1177/08862605241311611)
Supplement: sj-doc-2-jiv-10.1177_08862605241311611 – Supplemental material for Immigrant Background and Rape Conviction: A 21-Year Follow-Up Study in Sweden [file sj-doc-2-jiv-10.1177_08862605241311611.doc]

**Appendix 2 – Supplemental results**

*Table 1.* The five most common of origin among those born outside of Sweden.

| Country | *n* |
| --- | --- |
| Iraq | 500 |
| Somalia | 162 |
| Syria | 123 |
| Afghanistan | 100 |
| Iran | 99 |

*Table 2.* The three most common psychiatric diagnoses.

| Diagnoses | *n* |
| --- | --- |
| F19: Other psychoactive substance related disorders | 226 |
| F43: Reaction to severe stress, and adjustment disorders | 188 |
| F32: Depressive episode | 135 |

*Table 3.* The three most common prior offences.

| Country | *n* |
| --- | --- |
| Violent crimes | 1,660 |
| Property crimes | 1,282 |
| White collar crimes | 571 |
