# Supplementary material for: Immigrant Background and Rape Conviction: A 21-Year Follow-Up Study in Sweden
Source: J Interpers Violence. 2025 Jan 6;41(1-2):372–90. doi: 10.1177/08862605241311611 (PMC12662818; doi:10.1177/08862605241311611)
Supplement: sj-docx-1-jiv-10.1177_08862605241311611 – Supplemental material for Immigrant Background and Rape Conviction: A 21-Year Follow-Up Study in Sweden [file sj-docx-1-jiv-10.1177_08862605241311611.docx]

**Appendix 1 – Included Variables in the Study and their Definitions**

|  | Registers Used | Definition |
| --- | --- | --- |
| Psychiatric Disorder | The Swedish Hospital Discharge Register (coverage 1973-2020); Outpatient Care Register (national coverage 2001-2020); | ICD-8/9: 290-319; ICD-10: F00-F99. |
| Alcohol Use Disorder (AUD) | The Swedish Hospital Discharge Register (coverage 1973-2020); Outpatient Care Register (national coverage 2001-2020); the Swedish Drug Register (2005-2020); the Swedish Mortality Register, and the Swedish Criminal Register (1973-2020) and the Swedish Suspicion Register (1998-2020) | Alcohol Use Disorder (AUD) was identified in the Swedish medical and mortality registries by ICD codes: ICD9: V79B, 305A, 357F, 571A-D, 425F, 535D, 291, 303, 980; ICD 10: E244, G312, G621, G721, I426, K292, K70, K852, K860, O354, T51, F10); in the Swedish Criminal Register and the Swedish Suspicion Register with at least two registrations of drunk driving (suspicion code 3005, law 1951:649 (paragraph 4 and 4A)) or drunk in charge of a maritime vessel (suspicion code 3201, law 1994:1009 (chapter 20, paragraph 4 and 5)); in the Prescribed Drug Register by the drugs disulfiram (Anatomical Therapeutic Chemical (ATC) Classification System N07BB01), acamprosate (N07BB03), and naltrexone (N07BB04). |
| Drug Use Disorder (DUD)) | The Swedish Hospital Discharge Register (coverage 1973-2020); Outpatient Care Register (national coverage 2001-2020); the Swedish Drug Register (2005-2020); the Swedish Mortality Register, and the Swedish Criminal Register (1973-2020) and the Swedish Suspicion Register (1998-2020) | Drug abuse (DA) was identified in the Swedish medical and mortality registries by ICD codes (ICD8: Drug dependence (304); ICD9: Drug psychoses (292) and Drug dependence (304); ICD10: Mental and behavioral disorders due to psychoactive substance use (F10-F19), except those due to alcohol (F10) or tobacco (F17)); in the Suspicion Register by codes 3070, 5010, 5011, and 5012, that reflect crimes related to DA; and in the Crime Register by references to laws covering narcotics (law 1968:64, paragraph 1, point 6) and drug-related driving offences (law 1951:649, paragraph 4, subsection 2 and paragraph 4A, subsection 2). DA was identified in individuals (excluding those suffering from cancer) in the Prescribed Drug Register who had retrieved (in average) more than four defined daily doses a day for 12 months from either of Hypnotics and Sedatives (Anatomical Therapeutic Chemical (ATC) Classification System N05C and N05BA) or Opioids (ATC: N02A). |
| Criminal Behavior (CB) | The Swedish Criminal Register (1973-2020) and the Swedish Suspicion Register (1998-2020) | Any registration in the Criminal Register |
| Social welfare recipient | Longitudinal integrated database for health insurance and labour market studies (LISA) | Social assistance is defined as financial support under the Social Services Act. You can receive support for your upkeep and for other items that you need to have a reasonable standard of living. Examples of common situations when social assistance is given: As an income supplement to low-income families; for unemployed when other unemployment assistance is not provided or is insufficient; when sickness benefits are insufficient or not provided; to those who are bound by the children in the home and can not get childcare and therefore not can seek work. The variable is recorded at the family level, which means that all individuals in a family with social assistance will, in this report, be counted as recipients of Social welfare .  In our models we consider an individual as Social welfare recipient if they were registered the year prior to registration for rape+ |
| Neighborhood Deprivation | Longitudinal integrated database for health insurance and labour market studies (LISA), Register of Total Population | For every year an individuals is registered at a specific DESO area. The DeSO areas divides Sweden into 5,983 areas and have between 700 and 2,700 inhabitants. The division takes into account the geographical conditions so that the boundaries follow, for example, streets, waterways and railways. Important building blocks used to create DeSO are urban areas and electoral districts. For each of the DeSO area we created a neighborhood social deprivation (SD) index based on register data for all residents in the neighborhood aged 25-64. We used deprivation indicators used by past studies to characterize neighborhood environments and then used a principal component analysis to calculate a z-score. The following four variables were included: low educational status (defined as less than 10 years of formal education); low income (from all sources, including that from interest and dividends, which was defined as less than 50% of individual median income); unemployment (defined as not employed; excluding full-time students, those completing compulsory military service, and early retirees); and social welfare assistance. IN the models we include the Z-score as a continuous variable |
| Income | Longitudinal integrated database for health insurance and labour market studies (LISA) | Measured as the disposable income for the individual at the year prior to registration for rape+. In the models the variable is divided by 1000. |
